# CNF1 Improves Astrocytic Ability to Support Neuronal Growth and Differentiation *In vitro*

**DOI:** 10.1371/journal.pone.0034115

**Published:** 2012-04-16

**Authors:** Fiorella Malchiodi-Albedi, Silvia Paradisi, Michela Di Nottia, Daiana Simone, Sara Travaglione, Loredana Falzano, Marco Guidotti, Claudio Frank, Alessandro Cutarelli, Alessia Fabbri, Carla Fiorentini

**Affiliations:** 1 Department of Cell Biology and Neuroscience, Istituto Superiore di Sanità, Rome, Italy; 2 Department of Therapeutic Research and Medicines Evaluation, Istituto Superiore di Sanità, Rome, Italy; 3 Departmrent of Veterinary Public Health and Food Safety, Istituto Superiore di Sanità, Rome, Italy; 4 National Centre for Rare Diseases, Istituto Superiore di Sanità, Rome, Italy; University of Insubria, Italy

## Abstract

Modulation of cerebral Rho GTPases activity in mice brain by intracerebral administration of Cytotoxic Necrotizing Factor 1 (CNF1) leads to enhanced neurotransmission and synaptic plasticity and improves learning and memory. To gain more insight into the interactions between CNF1 and neuronal cells, we used primary neuronal and astrocytic cultures from rat embryonic brain to study CNF1 effects on neuronal differentiation, focusing on dendritic tree growth and synapse formation, which are strictly modulated by Rho GTPases. CNF1 profoundly remodeled the cytoskeleton of hippocampal and cortical neurons, which showed philopodia-like, actin-positive projections, thickened and poorly branched dendrites, and a decrease in synapse number. CNF1 removal, however, restored dendritic tree development and synapse formation, suggesting that the toxin can reversibly block neuronal differentiation. On differentiated neurons, CNF1 had a similar effacing effect on synapses. Therefore, a direct interaction with CNF1 is apparently deleterious for neurons. Since astrocytes play a pivotal role in neuronal differentiation and synaptic regulation, we wondered if the beneficial *in vivo* effect could be mediated by astrocytes. Primary astrocytes from embryonic cortex were treated with CNF1 for 48 hours and used as a substrate for growing hippocampal neurons. Such neurons showed an increased development of neurites, in respect to age-matched controls, with a wider dendritic tree and a richer content in synapses. In CNF1-exposed astrocytes, the production of interleukin 1β, known to reduce dendrite development and complexity in neuronal cultures, was decreased. These results demonstrate that astrocytes, under the influence of CNF1, increase their supporting activity on neuronal growth and differentiation, possibly related to the diminished levels of interleukin 1β. These observations suggest that the enhanced synaptic plasticity and improved learning and memory described in CNF1-injected mice are probably mediated by astrocytes.

## Introduction

Proteins belonging to the Rho GTPases' family, including Rho, Rac and Cdc42 subfamilies, act as molecular switches that cycle between a GDP-bound inactive and a GTP-bound active state to transduce extracellular signals to the actin cytoskeleton. Their ability to modulate the organization of the actin network [1] plays important roles in the morphogenesis of the dendritic spines of neurons in the brain [2], [3], [4] and synaptic plasticity [5], [6], [7], [8], [9], [10]. Although the picture is not fully resolved yet, it appears that Rac and Cdc42, which induce actin polymerization, meshwork formation and bundling, promote spine formation and maturation [11], whereas RhoA activation, which promotes actin contraction, results in spine retraction [12]. Dendritic spines are small, actin-rich protrusions, and actin dynamics regulates their shape and morphological plasticity. Importantly, activation of NMDA receptors (as occurs in LTP) affects dendritic spine morphogenesis by activation of Rac1 and actin remodeling [13], linking activity-dependent synaptic plasticity to Rho GTPases. In the nervous system, the Rho GTPases play a key role in several processes, and mutations in proteins involved in Rho GTPase signaling may be causative in some forms of mental retardation.

We have found that a bacterial protein toxin from *Escherichia coli*, which activates the Rho GTPases, can improve learning and memory in mice [14], [15]. This toxin, named cytotoxic necrotizing factor 1 (CNF1), acts by blocking the Rho GTPases in their activated, GTP-bound state by catalyzing the deamidation of a single glutamine residue of the Rho molecules, thus impeding GTP hydrolysis and leading to their persistent activation [16], [17]. CNF1 modulation of cerebral RhoA and Rac1 activity in mice re-arranges cerebral actin cytoskeleton, enhances neurotransmission and synaptic plasticity and improves cognitive performances [14], [15]. Also, CNF1 counteracts the formalin-induced inflammatory pain in mice, after both peripheral and central administration, further sustaining its ability in modulating CNS pathophysiology [18]. Very recently, we have demonstrated that CNF1, by directly modulating the brain Rho GTPases, triggers structural remodeling and functional plasticity into the adult rat visual cortex [19] and improves the behavioral phenotype in a mouse model of Rett syndrome [20].

Therefore, CNF1 can be viewed as a new pharmacological agent able to enhance the changes in neuronal connectivity associated with memory for by which the toxin can ameliorate the neuronal function. To address this question, we have performed a study on primary neuronal cultures of rat embryonic brain with the aim of investigating the effects of CNF1 on *in vitro* neuronal growth and differentiation, focusing on the development of dendritic tree and synapse formation. Our data show that while direct administration of CNF1 to neuronal cultures has a harmful effect on neuronal maturation, hippocampal neurons conditioned by CNF1-treated mation and to improve neuronal plasticity. It remains, however, to define the mechanisms astrocytes show an increase in dendrite growth and synapse formation, sustaining a role for CNF1 in improving astrocytic neurosupportive activity.

## Results

### CNF1 modifies neuritic tree and synapse development in neurons during differentiation

**Figure 1:**
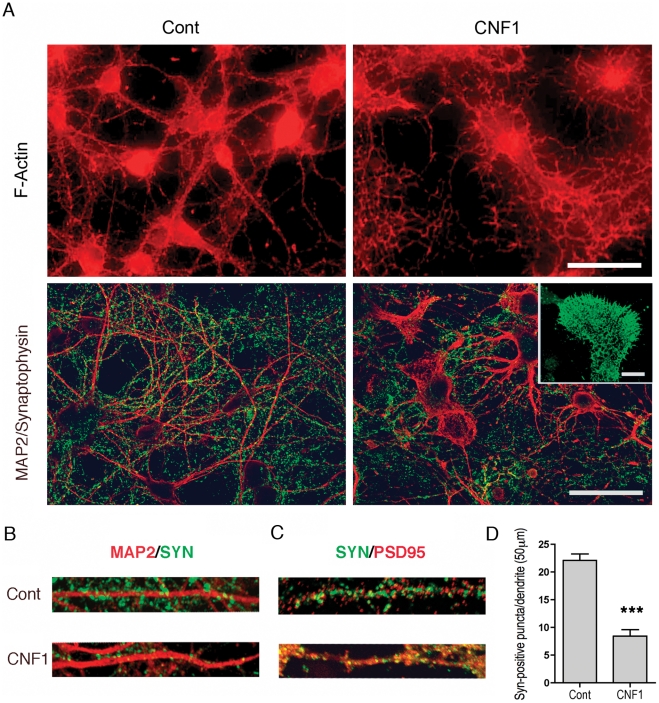
CNF1 modifies actin cytoskeleton and synapse development in hippocampal neurons. Pure hippocampal neurons were treated or not with CNF1 at DIV2 and fixed at DIV 14. A. Neurons were immunolabeled for F-actin, upper panel, or co-immunostained for synaptophysin (green) and MAP2 (red), lower panel. In mature (DIV 14) control neurons, actin-labeled neurites are long, thin and well defined, while after CNF1 treatment, the neuritic tree and the cell bodies have a spiny appearance, due to the presence of numerous short protrusions (bar  = 20 μm). In control cultures, at DIV 14, synaptophysin-positive synapses appear as small puncta, regularly distributed along the MAP2-positive dendrites. In CNF1-treated hippocampal cultures, fewer synaptophysin-positive dots can be observed along dendrites (bar  = 20 μm). A synaptophysin-labeled growth cone is visible in the inset (bar  = 10 μm). B. At higher magnification, the lower number of synaptophysin-positive puncta along dendrites, induced by treatment with CNF1, can be better appreciated. C. While in control neurons, PSD95 and synaptophysin are compartmentalized in the pre- and post-synaptic districts, respectively, in CNF1-treated neurons, the pre- and post-synaptic markers often co-localize and loose their dot-like appearance. D. Synapse density was measured as number of synaptophysin-positive puncta along dendrites, immunostained with MAP2. At least 30 images of dendrites (20 μm-long), obtained from 3 different cultures, were analyzed for each condition. The histogram represents the mean values ± S.E.M. *** = p<0.001 Statistical analysis was conducted by the nonparametric Mann-Witney U test.

To analyze the effects of CNF1 on neuronal differentiation, hippocampal cultures were treated at *day-in-vitro* (DIV) 2 with CNF1 and fixed at DIV 14. At this stage, staining with the nuclear dye Hoechst 33342 showed that, although cell density varied among different cell cultures, there was no significant difference in the neuronal cell number, within the same culture, between control and CNF1 treatment (data not shown). However, neuronal cell differentiation was profoundly affected. Indeed, while in mature control neurons actin-labeled neurites were long, thin and well defined, in CNF1-treated cultures, the neuritic tree and the cell bodies were covered with numerous and short protrusions, which gave the cells a spiny appearance (Figure 1A). CNF1-induced cytoskeletal changes were accompanied by a lack of synapse formation, as demonstrated by immunolabeling of synaptophysin, an integral protein of synaptic vesicles. In fact, in control cultures, at DIV 14, synaptophysin-positive synapses appeared as discrete dots, regularly distributed along the MAP2-positive dendrites (Figures 1A, B). In contrast, in CNF1-treated hippocampal cultures, synaptophysin immunolabeling was more dispersed and lacked the typical punctuated appearance along dendrites (Figure 1A, B). In addition, while in control neurons, PSD95, a marker of post-synaptic densities, and synaptophysin were separately compartmentalized in the pre- and post-synaptic districts, respectively, in CNF1-treated neurons, the pre- and post-synaptic markers often lost their dot-like appearance and co-localized (Figure 1C). Synaptophysin also showed a positivity in growth cones, which were frequently observed in CNF1-treated cultures (Figure 1A, inset). Counts of synaptophysin-positive puncta along dendrites confirmed a decrease in synaptic density in CNF1-treated cultures (Figure 1D).

**Figure 2:**
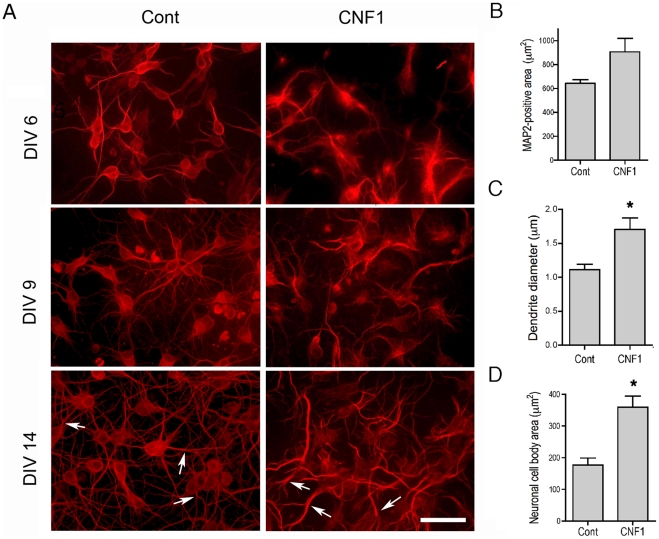
MAP2-labeling highlights CNF1-induced changes of the neuritic tree during differentiation. Pure hippocampal neurons were treated or not with CNF1 at DIV2, fixed at DIV 6, 9, and 14, and immunolabeled for MAP2 (Figure 2A). In control hippocampal neurons, MAP2-positive dendritic tree gradually enlarge and become ramified, until a complex network is formed. In neuronal cultures treated with CNF1, MAP2-positive dendrites become thicker, showing few ramifications. Neuronal cell bodies are larger, with a veil-like appearance (bar  = 20 μm). B, C, D. Morphometric analysis of MAP2-positive area, dendrite diameter and cell body area in hippocampal neurons at DIV 14, treated or not with CNF1. MAP2-positive, somatodendritic area (B) was measured and divided by the cell number in at least 5, randomly chosen fields (41.500 μm^2^) for each condition in 4 different cultures. Dendrite diameter was measured in at least 50 dendrites, just before the first dendritic branching. Measures obtained were averaged to produce a single mean value for each culture. Histogram shows mean values ± S.E.M of 4 different cultures. For neuronal cell body area, at least 15 MAP2-positive neuronal cell bodies were measured and averaged for each condition in 4 different cultures. The results show that the thickness of the dendrites and the cell body area are significantly increased in cells challenged with the toxin (* = p<0.05, Wilcoxon Matched Pairs test).

Labeling of MAP2, a marker of dendritic cytoskeleton, also highlighted CNF1-induced changes of the neuritic tree. In control pure hippocampal neurons, during differentiation, MAP2-positive dendritic tree gradually enlarged and became ramified, with thin and smooth projections, until a complex network was formed (Figure 2A, left column). Neuronal cell bodies maintained a round shape, with limited dimensions. When exposed to CNF1 from DIV 2 MAP2-positive dendrites appeared thicker and more tortuous. Thin ramifications were lacking. Neuronal cell bodies were larger, with a veil-like appearance (Figure 2A, right column). Morphometric analysis confirmed that the dendrite diameter (Figure 2C) and cell body area (Figure 2D) increased in cells challenged with the toxin. Total somatodendritic MAP2-positive area (Figure 2B), however, although showing a trend towards an increase, did not reach significant values in CNF1-treated cultures, possibly due to the loss of finer ramifications. Similar results were obtained in pure cortical neuronal cultures (data not shown).

### CNF1-induced changes in differentiating neurons are reversible

**Figure 3:**
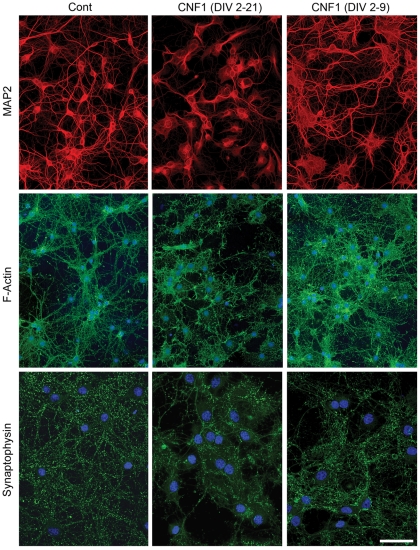
CNF1-induced changes in differentiating neurons are reversible. Neurons were either continuously exposed to CNF1 until DIV 21, or exposed to CNF1 until DIV 9 and then switched to CNF1-free medium until DIV 21. Control neurons received no treatment. Neurons were immunolabeled for MAP2 (red), actin (green) and synaptophisin (green). At DIV 21, neurons that had been exposed to CNF1 only until DIV 9 show less impressive changes than neurons exposed to CNF1 up to DIV 21. Dendrites are more numerous, although they are still thicker and fewer, compared to untreated cultures. Cell bodies also remain larger, with a veil-like appearance, but the actin cytoskeleton show the formation of thinner and longer projections. The number of synapses is increased. In micrograph showing F-actin and synaptophysin, nuclei are stained with DAPI (blue) (bar  = 50 μm).

In the experiments so far described, CNF1 was administered at DIV 2 and persisted in the medium until fixation. We asked if removal of the toxin during differentiation could modify the observed changes in the growth of neuritic tree and synapse formation (Figure 3). We compared the development of MAP2 positive dendritic tree, actin cytoskeleton and synaptophysin-labeled synapses in neurons continuously exposed to CNF1 until DIV 21, to neurons treated with CNF1 until DIV 9 and then switched to CNF1-free medium until DIV 21. At the end of the differentiation process, these neurons showed less impressive changes than neurons continuously exposed to CNF1. Dendrites increased in number, although they were still thicker and fewer, compared to untreated cultures. Cell bodies also remained larger, with a veil-like appearance, but the actin cytoskeleton showed the formation of thinner and longer projections. The number of synapses increased. These results suggest that block of maturation induced by CNF1 is partially reversible once the toxin is removed.

### CNF1 effects in mixed astrocytic/neuronal cultures

**Figure 4:**
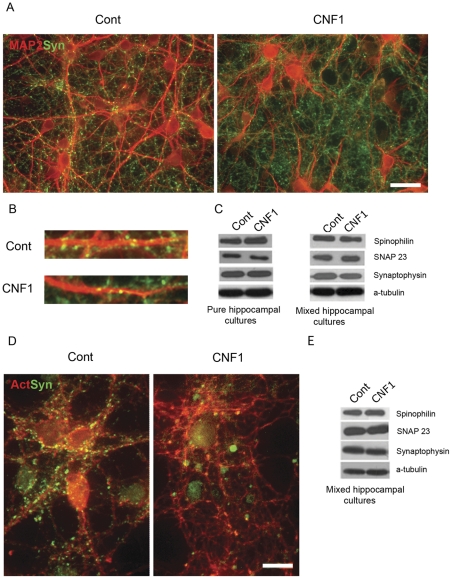
CNF1 effects in mixed astrocytic/neuronal cultures and in differentiated cultures. Cortical mixed astrocytic/neuronal cultures were treated with CNF1 at DIV2 and fixed at DIV 14. Neurons were immunolabeled for MAP2 (red) and synaptophysin (green) (A, bar  = 50 μm). In the presence of astrocytes, CNF1 induces less evident cytoskeletal changes than in pure neuronal cultures, with a minor dendritic tree remodeling, while a few synapses persist (Figure 4B). Western blot analysis, in both pure neuronal (Figure 4C, left) and mixed astrocytic/neuronal (Figure 4C, right) cultures, show that the levels of synaptic proteins, such as synaptophysin and SNAP23, or of components of the dendritic tree, such as spinophilin, are similar in CNF1-treated and control cultures. Differentiated neurons at DIV 12 were exposed to CNF1, fixed at DIV 14 and immunolabeled for actin (red) and synaptophysin (green) (Figure 4D, bar  = 20 μm). Under the influence of CNF1, actin cytoskeleton shows a marked remodeling, with increase in cell body size and development of fine and short neuritic branches. Synaptic density decreases, when compared to control cultures. Again, no changes in the expression of the synaptic proteins above mentioned was measured by Western blotting (Figure 4E).

Astrocytes have a pivotal role in neuronal differentiation. In several neuronal cell models, including hippocampal cultures, the presence of astrocytes improves and speeds up neuronal differentiation [21], [22]. We asked therefore if the presence of astrocytes could modify CNF1 effects on neuronal cell growth. At DIV14, in both hippocampal (data not shown) and cortical mixed astrocytic/neuronal cultures (Figure 4A, B), the presence of astrocytes rendered CNF1-induced neuronal cytoskeletal changes less evident than in pure neuronal cultures, with a minor dendritic tree remodeling (Figure 4A) and a decreased loss in synapses (Figure 4B). Still, neurons treated with CNF1 *in vitro*, even in the presence of astrocytes, had a less differentiated appearance than control cultures, a finding that could hardly contribute to explain the cognitive improvement observed after CNF1 treatment *in vivo*. Western blot analysis, conducted in both pure neuronal (Figure 4C, left) and mixed astrocytic/neuronal (Figure 4C, right) cultures, showed that the levels of synaptic proteins, such as synaptophysin and SNAP23, or of components of the dendritic tree, such as spinophilin, were similar in CNF1-treated and control cultures. This suggests that the observed changes in neuronal morphology in CNF1-treated cells were not due to a different expression of key molecules but probably depended on cytoskeletal remodeling.

### CNF1 effects in differentiated cultures

So far, we had analyzed neuronal cell development *in vitro* under the influence of CNF1 from the first stages of cell growth. We wondered if the toxin had different effects on differentiated neurons. For this purpose, we exposed to CNF1 neuronal cultures at DIV 12, a stage where polarity has been achieved and synapse formation is completed (Figure 4D). Again, under the influence of CNF1, actin cytoskeleton underwent a profound morphological remodeling. The cell body area increased and the neuritic network lost its typical structure, composed of long, thin and smooth neurites, and assumed a spider web-like appearance, with short, interconnected and irregular projections. This remodeling affected synapses, when compared to control cultures, as shown by decreased synaptophysin-positive dots. Yet, no changes in the expression of the synaptic proteins above mentioned could be measured (Figure 4E).

### CNF1-treated astrocytes provide a more efficient substrate to neuritogenesis and synaptogenesis

**Figure 5:**
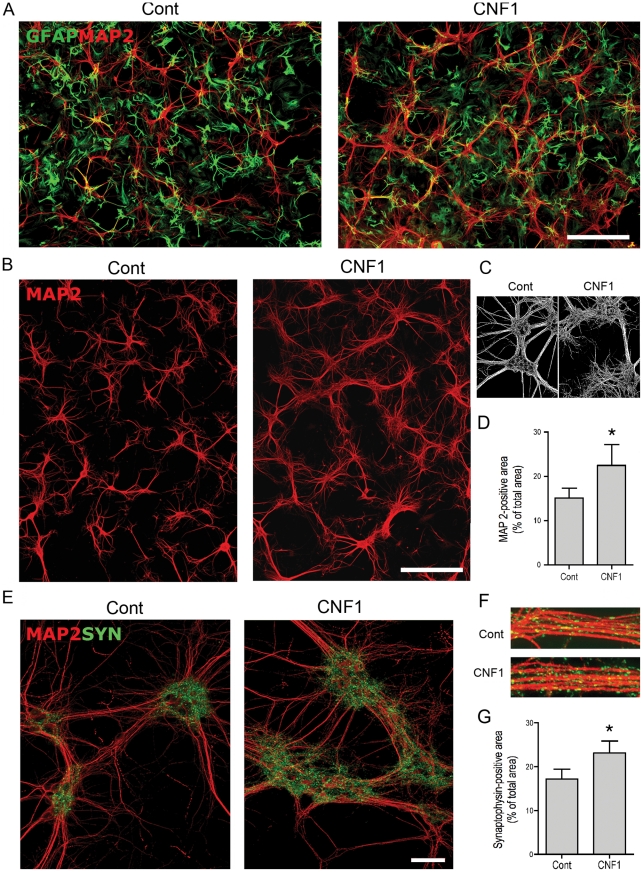
CNF1-treated astrocytes provide a more efficient substrate to neuritogenesis and synaptogenesis. Pure astrocytic cell cultures, at confluence, were treated with CNF1 for 48 h. After change of the medium, hippocampal neurons were seeded on the astrocytic monolayer, fixed at DIV 14, and immunolabeled for MAP2 (red) and GFAP (green) or synaptophysin (green). The exposure of astrocytes to CNF1 causes a decrease in GFAP staining (A) whereas hippocampal neurons, growing on CNF1-treated astrocytes but in absence of direct CNF1 influence, produce a much more abundant dendritic network, as shown by MAP2 immunolabeling (A,B, bars  = 200 μm). In C, a detail of a black and white image used for morphometric analysis emphasizes the change in the dendritic tree, which, growing on control astrocytes, appear smooth and poorly branching, while on CNF1-pretreated astrocytes, show much wider ramifications (C). D. Morphometric analysis of MAP2-positive area. In hippocampal neurons co-cultured with astrocytes, after background subtraction, MAP2-positive area was measured as percentage of the total field area (0.15 mm^2^). Values obtained for each field were pooled to obtain a single mean value for each neuronal culture (n = 5). Bars represent mean values ± S.E.M (Figure 5C, * = p<0.05, Wilcoxon Matched Pairs test). The richer dendritic tree is accompanied by an increased formation of synapses, as shown by synaptophysin immunolabeling, particularly enriched around cell bodies (E, bar  = 50 μm). Synaptophysin-positive puncta are also visible along dendrite bundles (F). G. Morphometric analysis of synaptic density. Synaptophysin-positive area was measured in 41.500 μm^2^-large fields obtained from 3 different experiments, conducted in duplicate from 2 different cultures. At least 16 images were analyzed for each condition and the results pooled. Histogram represents the values + S.E.M. Statistical analysis was conducted by the nonparametric Wilcoxon Matched Pairs tests.

In the protocols so far described, CNF1 was administered directly to neurons, in the presence or not of astrocytes. However, we reasoned that in *in vivo* treatment, where CNF1 is delivered by means of intracerebroventricular injections, the toxin first interacts with ependymal cells, which line the ventricles, and then with astroglial cells, which surround the ependymal layer. Thus we hypothesized that the beneficial effects observed *in vivo* could be mediated by the interaction of CNF1 with astrocyes. To address this question, we treated pure astrocytic cell cultures with CNF1 and analyzed how this treatment affected astrocytic ability to support neuronal cell growth. At a difference from the experiments so far conducted, hippocampal neurons, growing on CNF1-treated astrocytes, but in absence of direct CNF1 influence, produced a much more abundant dendritic tree, with richer branching, creating a confluent network, as shown by MAP2 immunolabeling (Figure 5A, B). At higher magnification, control dendrites appeared smooth and devoid of ramifications, while dendrites growing on CNF1-pretreated astrocytes showed an extensive web of fine projections (Figures 5C). Morphometric analysis confirmed the augmented MAP2-positive area (Figures 5D). Furthermore, in these neuronal/astrocytic co-cultures, immunolabeling for GFAP, an important component of the astrocyte cytoskeleton, was less evident in CNF1-treated astrocytes than in control cultures (Figure 5A). Interestingly, the enlargement of the dendritic tree was accompanied by an increased formation of synapses, as shown by synaptophysin immunolabeling (Figures 5E, F), which was particularly evident around neuronal cell clusters. Morphometric analysis of synaptophysin-positive area confirmed a significant increase of synapses in neuronal cell cultures growing on CNF1-pretreated astrocytes (Figure 5G).

### CNF1 treatment endows astrocytes with a neuroprotective phenotype

**Figure 6:**
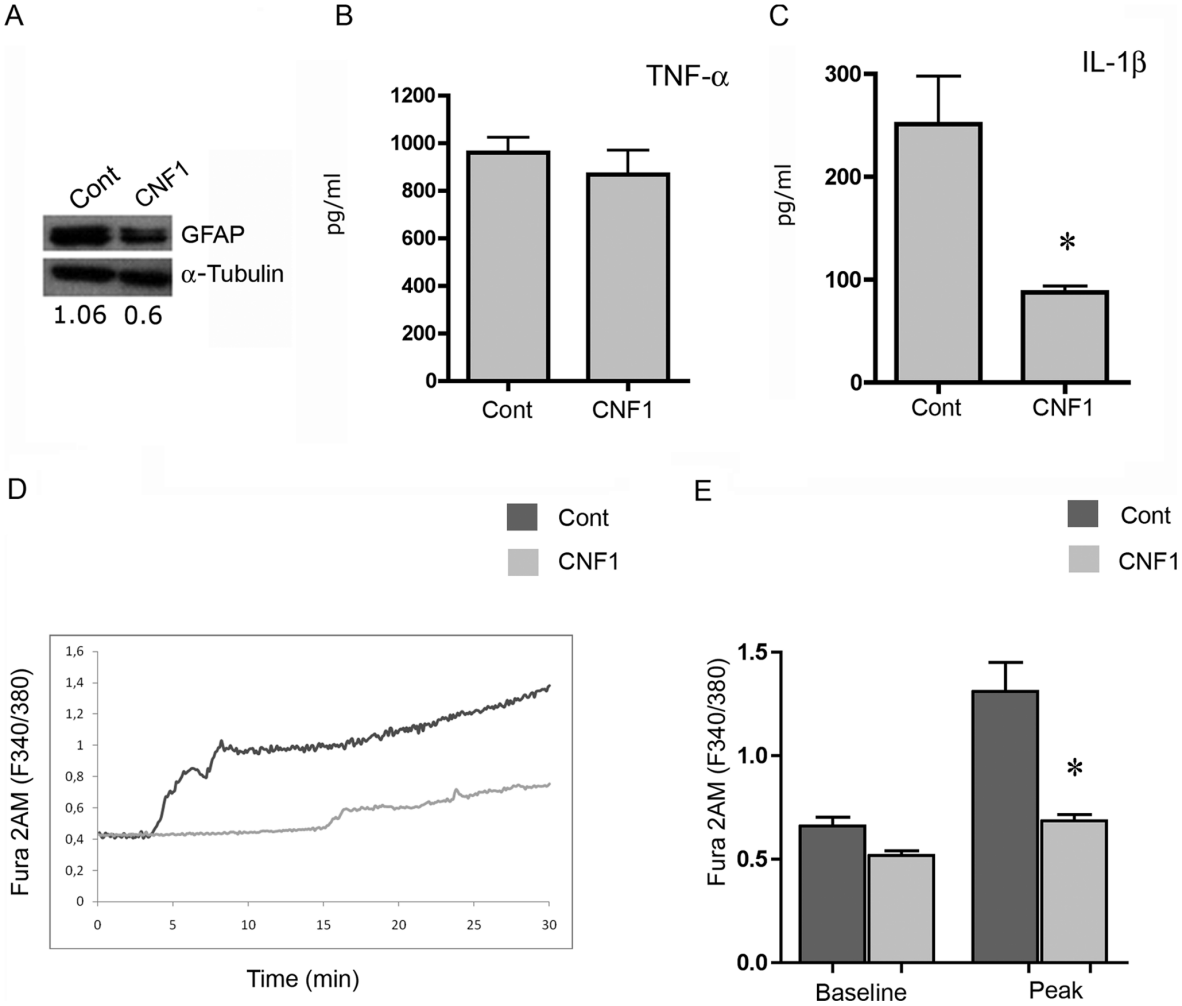
CNF1 treatment reduces GFAP and IL-1β levels and glutamate-dependent intracellular Ca^2+^ rise in pure astrocytic cultures. The CNF1-induced decrease in GFAP level, observed by immunofluorescence (Figure 5, panel A), is confirmed by Western blot analysis (panel A). In the supernatants from control and CNF1-treated astrocytes, the expressions of TNF-α and IL-1β were measured by ELISA. While the levels of TNF-α are unaffected (panel B), IL-1β is significantly decreased in astrocytes challenged with the toxin (panel C). Intracellular Ca^2+^ levels were analyzed in astrocytes, following administration of glutamate (after a 3 min baseline), using fluorimetric recordings with Fura 2 AM (panels D,E). In D, the time course of a representative experiment is shown. The values of at least 6 cells were recorded and averaged. In E, Ca^2+^ levels were compared at the baseline and in the peak region. Data for three different experiments were pooled and analyzed. At least 6 cells were evaluated in each experiment. Bars represent mean values ± S.E.M. Glutamate induces significantly lower Ca^2+^ peaks in astrocytes treated with CNF1 than in control cells (panels C and E *p<0.05 unpaired Student's t-test).

A reduction in GFAP content has been put in relation to increased astrocytic-induced dendritogenesis [23]. Therefore, to investigate the mechanisms by which CNF1 confers to astrocytes the above described properties, we grew pure astrocytic cultures and analyzed GFAP content by Western blotting. GFAP was evidently reduced after CNF1 treatment (Figures 6 panel A). Furthermore, in the same cultures, we measured the expression of the pro-inflammatory cytokines TNF-α and IL-1β after challenge with CNF1. We found that, whereas the expression of TNF-α was unaffected (Figure 6, panel B), IL-1β was significantly decreased in astrocytes challenged with the toxin (Figure 6, panel C). Since IL-1β directly impairs neurogenesis [24], its decrease is consistent with the observed positive modulation of dendritic growth after treatment with CNF1. Finally, we analyzed if CNF1 influenced the raise of intracellular Ca^2+^levels in astrocytes, following administration of glutamate, using fluorimetric recordings with Fura-2AM. We found that glutamate-induced Ca^2+^peaks were significantly lower in astrocytes treated with CNF1 (Figure 6D, E), suggesting the induction of a more resistant phenotype to gliotoxicity.

## Discussion

Astrocytes, the most abundant glial cells in the brain, have long been considered as a mere trophic support for neurons in the CNS. Recently, however, several studies have highlighted their importance in functions such as neurogenesis, neurotransmission, metabolite and electrolyte homeostasis, synaptogenesis and synapse modulation [25]. In this paper, we show for the first time that astrocytes challenged with the Rho-activating CNF1 provide a more efficient substrate to neuronal growth in primary neuronal cultures. Also, we herein demonstrate that a direct exposure of neurons to CNF1 leads to a reversible block of their maturation, supporting the hypothesis that astrocytes but not neurons are pivotal in the enhanced neurotransmission and synaptic plasticity we previously observed after *in vivo* treatment with CNF1 [14], [15].

The improvement of learning and memory that follows the intracerebral administration of CNF1 suggests that the toxin can influence the physiology of CNS by modulating Rho GTPases, which play a fundamental role in the development of dendritic spines and synapses [26], [27], [28]. To closely monitor CNF1 effects on neuronal growth and differentiation, in search of a morphologic counterpart of the improvement in learning and memory of CNF1-treated mice, we exposed to CNF1 primary hippocampal cultures from rat embryonic brains. Neurons treated with CNF1, especially from the early stages of development, underwent profound modifications, including the development of filopodia-like actin-positive protrusions along neurites and around cell bodies, thickening of dendrites, lack of synapse formation, poor dendritic branching. Similar results were obtained in the presence of astrocytes or for neuronal cultures obtained from a different brain region, such as the cortex. Analogous, although less evident, effects were observed after CNF1 treatment of mature neurons. Synaptic loss was not due to decreased synthesis of proteins of synaptic vesicles, whose levels were similar to that of control cultures, as shown by Western blot experiments. Thus, far from enriching the neuritic network as expectable from *in vivo* results, CNF1-treated neurons reduced the complexity of dendritic tree with respect to control neurons and, furthermore, inhibited the formation of synapses. This pattern of growth suggested a block of neuronal maturation, which was also suggested by the persistence of growth cones, normally disappearing after the first days in culture, in advanced stages of development (DIV 14). Consistently, removal of the toxin from the cell culture medium allowed the neuronal cultures to regain the differentiation process, with recovered growth of dendrite network and synapse formation. The results of these experiments, where CNF1 was administered directly to neurons, in the presence or not of astrocytes, did not explain the beneficial effects of CNF1 on behavioral tasks observed in *in vivo* administration of the toxin. However, we reasoned that in *in vivo* treatment, where CNF1 is delivered by means of intracerebroventricular injections, the toxin first interacts with ependymal cells, which line the ventricles, and then with astroglial cells, which are in close contact with the ependymal layer. Thus we hypothesized that the effects observed *in vivo* could be mediated by the interaction of CNF1 with astrocyes. To address this issue, we analyzed the growth of hippocampal neurons on astrocytes that had been previously treated with CNF1. Intriguingly, when seeded on CNF1-treated astrocytes, neurons showed a much more abundant development of dendritic tree, as compared to neurons grown on untreated astrocytes. Furthermore, synapses developed normally and were more numerous. It is reasonable to speculate that the enhanced synaptic plasticity and improved learning and memory observed *in vivo* could be mediated by the interaction of CNF1 with astrocyes.

It has long been known that astrocytes, when co-cultured with neurons, have the ability to improve neuronal growth, differentiation and synaptic formation [29]. How did CNF1 improve this ability? First of all, we found that the toxin induced a decrease in GFAP expression. GFAP is the main intermediate filament protein in mature astrocytes, but also an important component of the cytoskeleton in astrocytes during development. GFAP has been shown to be involved in astrocyte functions relevant to CNS regeneration and synaptic plasticity. Several lines of evidence suggest that the observed reduction in GFAP content in CNF1-treated astrocytes could be related to the increased dendritogenesis. GFAP has in fact been found to be a negative regulator of astrocytic ability to improve neuronal growth and neuritogenesis [30]. In addition, highly reactive astrocytes, as shown by GFAP immunostain, induce the formation of fewer synaptic contacts in co-cultured neurons, compared to less reactive astrocytes [31]. Recent studies showed that increased astrocytic GFAP expression can be related to neuron atrophy whereas diminished GFAP content restores neurite outgrowth in certain conditions [32]. It is worthwhile mentioning that various pathologic conditions of CNS are accompanied by reactive gliosis, which is characterized by an increase in the expression of GFAP and is considered to have a role in neurodegeneration [23]. Therefore, the capacity of CNF1 of modulating GFAP content could result crucial in those neurological diseases where astrocytosis contributes to neuronal damage.

It is well known that in GFAP-overexpressing, activated astrocytes, the secretion of pro-inflammatory cytokines is up-regulated and it is believed to contribute to neurodegeneration [33]. We wondered if CNF1-treated astrocytes, where low levels of GFAP are detected, also showed decreased levels of cytokines. In our model, when exposed to CNF1, astrocytes reduced the secretion of IL-1β while that of TNF-α was unvaried, suggesting a selective effect of CNF1 on the cytokine network. This result is in line with our results, since IL-1β can significantly reduce dendrite development and complexity in neuronal cultures [34]. In addition, upregulation of IL-1β was observed to negatively influence neurogenesis [24] and neurodevelopment [35], possibly by interfering with the signaling of BDNF, a major trophic factor in the CNS, critical for the development and survival of certain neuronal populations [36]. Thus, it is reasonable to expect that low levels of this cytokine may be related to an improvement of neuronal growth. In addition, it is worth noting that IL-1β is considered to contribute to neurotoxicity in several neurodegenerative diseases. This observation further prompts to consider CNF1 as a candidate therapeutic option in neurodegenerative conditions where upregulation of proinflammatory cytokines is considered of pathogenetic relevance.

Like in neurons, overstimulation of glutamate receptors induce in astrocytes an increase in intracellular Ca^2+^concentrations, which may be at origin of gliotoxic effects, resulting in dysfunction and death of astrocytes [37]. Since astrocytes are the major sink of glutamate in the brain, being mainly responsible for glutamate removal from the extracellular space [38], they represent one of the important components of CNS defense against glutamate excitotoxicity. For this reason, dysfunctional astrocytes may exacerbate excitotoxic cell damage [39]. We checked if CNF1 treatment could modulate glutamate response in astrocytes and found that intracellular Ca^2+^peak, following glutamate administration, was significantly reduced, compared to untreated astrocytes, suggesting an increased Rho-dependent ability of astrocytes to counteract excitotoxic stimuli.

As a whole, these results suggest that the changes observed in astrocytes after CNF1 treatment (decrease in GFAP and IL-1β levels, reduction of glutamate-driven intracellular Ca^2+^concentrations) i) provide a rationale to the observed improvement of neuritic tree growth in co-cultured neurons; ii) depict a neuroprotective astrocytic phenotype. In conclusion, considering the pathogenetic link between astrocytes and neurological disorders, through pathways that include inflammation, oxidative stress and excitotoxicity [40], our results encourage further studies on CNF1-astrocyte interactions, also in view of a possible development of new therapeutic approaches.

## Materials and Methods

### Ethics Statement

All primary cultures used in this study were obtained from Wistar rat embryos at gestational day 18 (Charles River). This study was carried out in strict accordance with the recommendations in the Guide for the Care and Use of Laboratory Animals of the National Institutes of Health. The protocol was approved by the Committee on the Ethics of Animal Experiments of the University of Minnesota (Permit Number: 27-2956).

### Primary cultures

After dissection, hippocampi were dissociated in trypsin and plated on poly-L-lysine-coated glass coverslips in Minimum Essential Medium (MEM), containing 10% fetal calf serum; after two hours, the medium was replaced with Neurobasal Medium (NBM) supplemented with B27. To obtain pure neuronal cultures, hippocampal neurons were treated at day-*in-vitro* (DIV) 1 with 1.5 μM Arabinosyl-Cytosine (Ara-C). In these conditions, neuronal cultures contain 1–2% of Glial Fibrillary Acidic Protein-positive astrocytes [41]. To analyze neuronal cell number, nuclei were stained with Hoechst 33342 in pure neuronal cell cultures at DIV 14. Five different fields (20x) were randomly chosen in coverslips obtained from four different cultures. Nuclei were counted and averaged. To obtain mixed neuronal/astrocytic cultures, no Ara-C was added. At DIV 14, in mixed cultures astrocyte number ranged from 20 to 40% of the cell population (data not shown). For pure and mixed cortical cultures, cortices were dissected, dissociated and plated, as described for hippocampal cultures. In all neuronal cultures (with the exception of cultures undergoing CNF1 interrupted treatment, see below), cell culture media were never totally substituted and small aliquots of NBM-B27 (5–10% in volume) were added once a week. In the protocol for CNF1 interrupted treatment, where CNF1-containing cell culture medium was substituted with CNF1-free medium at DIV 9, NBM-27 conditioned by untreated cultures grown in parallel was used. Primary astrocytic cultures were obtained from the cortex of rat embryos. After dissection and dissociation, as already described, cortical cell suspension was seeded in flasks in MEM, containing 10% fetal calf serum and let grow until confluence. Cells were replated twice to obtain a cell culture highly enriched in astrocytes. Contamination of microglial cells was below 1%, as shown by staining with *Bandeiraea simplicifolia* lectin-peroxidase conjugate (data not shown). For primary astrocytic-neuronal co-cultures, astrocytes were first seeded on glass coverslips and let grow until confluent. Hippocampal neuron suspension, obtained as described above, was seeded on the astrocytic monolayer and treated at DIV 1 with Ara-C, to block further growth of astrocytes. All cell cultures were grown at 37° in 5% CO_2_.

### CNF1 preparation and treatments

CNF1 was obtained from the 392 ISS strain (kindly provided by V. Falbo, Rome, Italy) and purified as previously described [42]. CNF1 was used at the concentration of 10^−10^ M.

Cell cultures were treated according different protocols. A) Treatment of immature neurons: CNF1 was administered to pure or mixed neuronal cultures at DIV2 until fixation (5 or 9 or 14 or 21 DIV). B) Treatment of mature neurons: hippocampal neurons at DIV 12 were treated for 48 h and then fixed. C) Interrupted treatment of immature neurons: pure hippocampal neurons were treated at DIV 2; at DIV 9, CNF1-containing medium was substituted with CNF1-free medium. At this stage of development, neuronal survival deeply depends on autocrine production of growth factors, thus abrupt change of medium with fresh NBM-B27 is deleterious for neurons *in vitro*. To overcome this problem, NMB-B27 derived from untreated cultures grown in parallel was used instead of fresh medium. Hippocampal cultures were fixed at DIV 21. D) Treatment of astrocytes to be co-cultured with neurons: confluent primary astrocytic cell cultures were treated for 48 h with CNF1. At the end of treatment, CNF1-containing medium was changed with CNF1-free NBM-B27 and primary hippocampal neurons were seeded on the astrocytic monolayer. In control cultures, hippocampal neurons were seeded on untreated astrocytes. Neuronal-astrocytic co-cultures were fixed at DIV 14.

### Immunocytochemistry

Cell cultures were fixed in 4% paraformaldehyde in phosphate buffered saline (PBS), 0.12 M in sucrose, and permeabilized with Triton X-100 (0.2%, Sigma). For F-actin detection, cells were stained with FITC (fluoresceine isothyocianate)-phalloidin (Sigma; working dilution 0.5 μg/ml in PBS) for 30 min at 37°C. Immunostaining was performed with the following primary antibodies: anti-microtubule-associated protein MAP2, a marker of dendrites, anti-synaptophysin, a synaptic vesicle-associated protein, PSD95, a marker of post-synaptic densities, glial fibrillary acidic protein (GFAP), specifically identifying astrocytic cytoskeleton. All primary antibodies were purchased from Millipore, MA, USA. After washing, samples were double-labeled with anti-mouse Alexa Fluor 488 and anti-rabbit 594 (Molecular Probes). In some experiments, nuclei were counterstained with DAPI or Hoechst 33342. Finally, after extensive washes, samples were mounted and observed with an Olympus BX51 fluorescence microscope or an Eclipse 80i Nikon Fluorescence Microscope, equipped with a VideoConfocal (ViCo) system.

#### Morphometric analysis

Morphometric analysis was conducted with the Optilab software (Graftek, Austin, TX) on images obtained at the Nikon Fluorescence Microscope, equipped with the ViCo system. In MAP2-immunostained, pure hippocampal neurons at DIV 14, mean somatodendritic area, mean dendritic diameter and mean cell body area were measured in control and CNF1-treated neurons. For somatodendritic area, total MAP2-positive area was measured and divided by the cell number in at least five, randomly chosen fields (41.500 μm^2^) for each condition in 4 different cultures. Dendrite thickness was measured before the first dendritic branching. At least 60 dendrites were randomly chosen from two separate coverslips of the same culture, measured and averaged, to produce a single mean value for each condition in 4 different cultures. For neuronal cell body area, at least 15 MAP2-positive neuronal cell bodies were measured and averaged for each condition in 4 different cultures. For synaptic density, synaptophysin-positive puncta were counted along MAP2-positive dendrites. At least 30 images of dendrites (20 μm-long), obtained from 3 different cultures, were analyzed for each condition. In hippocampal neurons co-cultured with astrocytes, after background subtraction, MAP2-positive area was measured as percentage of the total field area. In this case, positive area could not be normalized by cell number, since in neuronal/astrocytic co-cultures neurons grow in tight clusters and single cells are difficult to identify. Values obtained for each field (0.15 mm^2^) were pooled to obtain a single mean value for each neuronal culture (n = 5). A different procedure was also used to measure synaptic density. Again the reason was the peculiar growth modality of neuronal/astrocytic co-cultures, where dendrites develop as tightly packed bundles and single dendrites are difficult to discern. This did not allow counting synapses along dendrites, as performed in pure neuronal cultures. In addition, in neuronal/astrocytic co-cultures, synapses are particularly enriched around neuronal cell bodies, away from dendrites. For these reasons, synapse density was measured as synaptophysin-positive area. At least 16 images (41.500 μm^2^-large fields), obtained from 3 different experiments, conducted in duplicate from 2 different cultures, were captured and measured for each condition and the results pooled.

Statistical analyses were conducted by the nonparametric Mann-Witney U or Wilcoxon Matched Pairs tests.

### Western blot analysis

Cells were lysed in boiled sample buffer 1x (50 mM Tris-HCl, pH 6.8, 2% SDS, 10% glycerol, and 100 mM dithiothreitol). Twenty-five micrograms of total protein extracts were resolved by SDS-polyacrylamide gel electrophoresis (PAGE) and electrically transferred onto polyvinylidene difluoride membranes (Bio-Rad). Membranes were blocked with Tris-buffered saline-Tween 20 (TBS-T) (20 mM Tris-HCl, pH 7.4, 150 mM NaCl, and 0.02% Tween 20) containing 5% skimmed milk (Bio-Rad) for 30 min at room temperature, and then they were incubated overnight at 4°C with primary antibodies diluted in TBS-T containing 2% milk. The following primary antibodies were used: mouse monoclonal anti-synaptophysin (Chemicon; 1∶1000), rabbit polyclonal anti-spinophilin (Upstate; 1∶1000), mouse monoclonal anti-SNAP-23 (Sy-Sy; 1∶10000), rabbit polyclonal anti-GFAP (Millipore; 1∶5000), mouse monoclonal anti-alpha-tubulin (Sigma; 1∶10000). After extensive washing, immune complexes were detected with horseradish peroxidase-conjugated species-specific secondary antibodies (Jackson's) followed by enhanced chemiluminescence reaction (Amersham).

### Enzyme-linked immunosorbent assay (ELISA)

For detecting IL-1β and TNF-α, ILs ELISA kits were used following the manifacturer's instructions (BioVendor-Laboratorni, a.s.). Briefly, after having washed the microtiterplate with Wash Buffer, 50 µl of each sample were pipetted in duplicate to the sample wells. After the Biotin-Conjugate addition to all wells and a subsequent Streptavidin-HRP incubation, the plate was carefully washed and the chromogenic substrate solution was added. Following appropriate incubation, the enzymatic reaction was stopped and the absorbance was red on a spectro-photometer (BIORAD) using 450 nm as the primary wave length, with a sub-wavelength of 650 nm.

### Fura-2AM experiments

Experiments were performed on astrocytes treated with CNF1 for 72 h. Optical fluorimetric recordings with Fura-2AM were used to evaluate the intracellular calcium concentration ([Ca_2_^+^]i). Fura-2AM stock solutions were obtained by adding 50 μg of Fura-2AM to 50 μl of 75% DMSO plus 25% pluronic acid. Cells were bathed for 60 min at room temperature with 5 μl of stock solution diluted in 1 ml of extracellular solution (in mM: 125 NaCl, 1 KCl, 5 CaCl2, 1 MgCl2, 8 glucose, and 20 HEPES, pH 7.35) for a final Fura concentration of 5 μM. This solution was then removed and replaced with extracellular solution, and the dishes were quickly placed on the microscope stage. To measure fluorescence changes, a Hamamatsu (Shizouka, Japan) Argus 50 computerized analysis system was used, recording every 6 s the ratio between the values of light intensity at 340 and 380 nm stimulation. The basal level of [Ca_2_^+^]i was estimated as approximately 80 nM using the calibration standard kit (Molecular Probes), equivalent to a ratio value of about 0.8.
